# COVID-19 Pandemic Impacts on the Mental Health of Professional Soccer: Comparison of Anxiety Between Genders

**DOI:** 10.3389/fpsyg.2021.765914

**Published:** 2021-11-11

**Authors:** Natã Sant’Anna Esteves, Michele Andrade de Brito, Vanessa Teixeira Müller, Ciro José Brito, Diego Ignacio Valenzuela Pérez, Maamer Slimani, Nicola Luigi Bragazzi, Bianca Miarka

**Affiliations:** ^1^Laboratory of Psychophysiology and Performance in Sports and Combats, Postgraduate Program in Physical Education, School of Physical Education and Sport, Federal University of Rio de Janeiro, Rio de Janeiro, Brazil; ^2^Postgraduate Program in Physical Education, School of Physical Education and Sports, Federal University of Juiz de Fora, Juiz de Fora, Brazil; ^3^Escuela de Kinesiología, Facultad de Salud, Universidad Santo Tomás, Santiago, Chile; ^4^Magister en Ciencias la Actividad Física y Deportes Aplicadas al Entrenamiento Rehabilitación y Reintegro Deportivo, Universidad Santo Tomás, Santiago, Chile; ^5^Section of Psychiatry, Department of Neuroscience, Rehabilitation, Ophthalmology, Genetics, Maternal and Child Health, Genoa University, Genova, Italy; ^6^Laboratory for Industrial and Applied Mathematics, Department of Mathematics and Statistics, York University, Toronto, ON, Canada

**Keywords:** coronavirus, sport psychology, team sport, mood state, gender, psychiatry, fear

## Abstract

This study verifies associated factors with trait and state anxiety in professional soccer teams during the COVID-19 pandemic. The sample was composed of 315 athletes, coaches, and physical trainers of professional soccer teams during the COVID-19 pandemic. From this amount, 214 were classified with trait anxiety, and 315 were classified with state anxiety using the State-Trait Anxiety Inventory (STAI). This study is an epidemiological and cross-sectional study. We applied an observational method, and we performed a remote measurement. The measurement was made via online questionnaires in male and female individuals working on soccer teams (soccer professionals or athletes) who could be affected by anxiety during social isolation in the COVID-19 pandemic. Each questionnaire was composed of sociodemographic questions, self-perceived performance, and STAI. The main results indicated a significant difference between female vs. male soccer professionals in state anxiety (54.97 ± 9.43 vs. 57.65 ± 9.48 index) and trait anxiety (54.21 ± 5.74 vs. 55.76 ± 6.41 index) with higher results in men. Sociodemographic variables impacted significant differences between female and male athletes and professionals of soccer clubs, and anxiety during the pandemic COVID-19 period impacted self-perceived performance analysis. The present results highlight the importance of cognitive behavior therapy for professional soccer teams.

## Introduction

COVID-19 was discovered in a Chinese province called Wuhan, and it is a virus in the family of coronaviruses responsible for respiratory diseases. Every year, thousands of people are infected with a common virus of this classification, causing flu symptoms (WHO, 2020). Unlike standard versions of the coronavirus, COVID-19 is much more aggressive and fatal to the human organism, according to the Ministry of Health of Brazil (2020). Due to the lack of knowledge about the virus and its mutations, no drug treatment guarantees the cure or prevention of the disease. The mobilization to face this disease is global, and research in the area increases its understanding. As it is a disease with a high rate of transmission, containment measures had to be taken in order to minimize the spread and its associated damage, such as the use of masks, constant hand washing, and more significant concern with hygiene practices, prohibitions of circulation, and suspension of events of all magnitudes, including sports (WHO, 2020). Staying at home has become the new routine in Brazil and the world. However, being at home in a time of so much uncertainty regarding the virus and humanity’s future can lead to problems such as anxiety because the changes in habitual ways of life can make people feel anxious and insecure (Usher et al., 2020). Therefore, the need for social support is more significant in times of adverse situations and events, then cutting social support as part of an imposed quarantine or isolation strategy can threaten the individual with the loss of the sense of connection and can have a considerable impact on mental health (Hawryluck et al., 2004). Anxiety is a feeling characterized by a concern for future things, someone’s desires to accomplish something, or experience a situation they are not aware of Allen et al. (1995). However, we cannot always face anxiety as a bad thing, since in mild degrees, being anxious can serve as a motivation to perform a football task, for example.

On the other hand, repetitive anxiety and long duration should turn on a warning signal. Suppose the stress response generates frequent and lasting or intense physiological activation. In that case, it can precipitate a depletion of the subject’s resources with the appearance of various psychophysiological disorders, which may predispose to the appearance of anxiety disorders among other mental disorders (Margis et al., 2003). Among the existing forms of anxiety, we can define it with two concepts: state-anxiety, referring to a transient emotional state, characterized by subjective feelings of tension that can vary in intensity over time, and trait anxiety, which refers to a relatively stable personal disposition to respond with anxiety to stressful situations and a tendency to perceive a more significant number of situations as threatening (Spielberger et al., 1970; Margis et al., 2003; Gama et al., 2008; Weinberg and Gould, 2018). These types of anxiety can be scientifically measured by STAI (State-Trait Anxiety Inventory).

Anxiety is present in all individuals to different degrees, either when the person is classified as someone with trait anxiety or state anxiety, which shows that the same condition will not affect two people equally. For example, women are more susceptible to risk than men of developing anxiety disorders throughout their lives. Several studies suggest a greater severity of symptoms, greater chronicity, and more significant functional impairment of women’s anxiety disorders. According to previous studies, the probable causes of this difference between genders are the genetic factors and the influence exerted by female sex hormones (Kinrys and Wygant, 2005). Animal models and some human research suggest that progesterone may be associated with anxiety. As progesterone changes naturally throughout the menstrual cycle, the increase in the hormone in women may be related to increased anxiety (Reynolds et al., 2018). The difference between genders is not the only comparison and understanding anxiety in sport can be substantial. Anxiety is inherent in the sport, as it can be experienced at all levels (Ford et al., 2017; Ayuso-Moreno et al., 2020). Because of this, we can find several terms of anxiety when we relate anxiety to sport (Patel et al., 2010). However, in the context of the COVID-19 pandemic, it seems likely that there will be substantial increases in anxiety and depression, substance use, the loneliness that will undoubtedly have consequences for mental health and well-being, both in the short and long term (Galea et al., 2020). In soccer, professionals from all departments and athletes may be suffering from the threat of COVID-19, the reality of social isolation, and uncertainties about the situation and its future. Because experiencing a stranger due to a perceived lack of information contributes to uncertainty, which can impair an individual’s ability to prepare effectively and efficiently for the future and thus increase vulnerability to symptoms related to anxiety (Grupe and Nitschke, 2013; Carleton, 2016; Chen et al., 2018; Galea et al., 2020).

COVID-19 is an entirely new tension event, and the need for quarantine is a reality that few generations have lived through. According to Usher et al. (2020), for many people, daily life changes dramatically, and “normal” ways of life, as we know them, are suspended indefinitely. Quarantine or imposed isolation is an unknown and unpleasant experience that involves separating friends and family and a departure from typical routines in everyday life. So, soccer players and professionals involved in the sport tend to be anxious due to the drastic break from routine, which may generate a feeling that there is nothing more to do. Besides, the uncertainties with the return, conditions for a safe return for them and their families, and the risk of losing their jobs bother professionals and players who depend on soccer’s livelihood. Therefore, the study’s objective is to compare the factors that can increase trait and state anxiety among professionals and soccer players according to the influence of gender during the COVID-19 pandemic.

## Materials and Methods

### Study Design

This study is an epidemiological and cross-sectional study. We applied an observational method, and we performed a remote measurement. The measurement was made via online questionnaires in male and female individuals working on soccer teams (soccer professionals or athletes) who could be affected by anxiety during social isolation in the COVID-19 pandemic. Each questionnaire was composed of sociodemographic questions, self-perceived performance, and State-Trait Anxiety Inventory (STAI). The measurement was conducted for 20 days in May 2020. This was the period that the epidemic curve was growing in Brazil, and the authorities were debating the return of soccer in the country.

### Participants

The sample was composed of 563 athletes, coaches, and physical trainers of professional soccer teams during the COVID-19 pandemic. When we separate the sample by gender and occupation, we had 464 men (294 = soccer professionals and 170 = athletes) and 99 women (28 = professionals and 71 athletes) participated in the study. These athletes were from professional soccer teams of 24 different states (São Paulo, Sergipe, Rio de Janeiro, Santa Catarina, Rio Grande do Sul, the Rio Grande do Norte, Piaui, Goiás, Mato Grosso, Mato Grosso do Sul, Minas Gerais, Paraná, Maranhão, Paraíba, Pernambuco, Pará, Rondônia, Bahia, Amazonas, Espírito Santo, Tocantins, Ceará, Alagoas, Acre) in Brazil, continuously competing in state, national and international representative championships with regular training (technical and tactical) 4–7 times a week before the COVID-19 pandemic. They performed basic physical training under 75% of maximal heart rate ∼3 times a week during the quarantine. The inclusion criteria for the present research included participants aged over 18 years without cognitive alterations, without surgeries or injuries, and having played more than 3 years in the professional soccer level, without COVID-19 or positively contagious but in an asymptomatic situation and discharged by a doctor. The present study had as exclusion criteria soccer athletes, coaches, and physical trainers who were unable to respond to the questionnaire or who had limitations during the study, mainly for health reasons, duly certified by doctors. Also, the participants were instructed not to intake alcohol or drugs for at least 24 h before the measures and were maintaining typical diets.

Before proceeding with data collection, all participants attended a briefing meeting and signed an informed consent document to ensure the understanding of the testing parameters and the risks and benefits associated with the study. This study was submitted to and approved by the Local Committee of Ethics in Research (no. 68598317.0.0000.5147), following the rules of resolution of the National Health Council and according to the WMA Declaration of Helsinki.

### Procedures and Measures

The present research is a cross-sectional epidemiological study carried out from May 12, 2020 to June 3, 2020, and the collection took place during the COVID-19 pandemic. The sample of this study consisted of athletes and adult professionals of both genders and comprised a set of self-reported questionnaires evaluating different domains of behavior and feelings of an individual in relation to the period of isolation of COVID-19. Google forms were used as a search platform for electronic distribution. it was limited to one answer per account, removing the duplicates. Respondents were required to log in to Google. Social media were used to advertise and recruit potential volunteers. Participants also completed a consent form reflecting the confidential and voluntary nature of their involvement in the study. The following were applied: Sociodemographic questions, Self-Perceived Performance Scale, State-Trait Anxiety Inventory.

#### Sociodemographic Questions

The present study applied sociodemographic questions in participants with the variables: gender, age, schooling level, club state, soccer experience, the average number of official games by month, experience in soccer (years), tournament experience, level of the championship (Solleveld et al., 2015; Kristjánsdóttir et al., 2019).

#### Self-Perceived Performance Scale

The SPS was adapted from preceding authors and intended to describe self-perceived performance (Gómez-Millán et al., 2017; Núñez et al., 2020). This assessment uses the theory of self-efficacy with the judgments that individuals have about themselves. The athlete had to indicate the frequency with which each one of the characteristics occurs in the proposed situation according to a Likert scale of 7 points (e.g., self-performance until now, physical, technical, tactical, psychological, disciplinary, and the self-performance in a team) (Gómez-Millán et al., 2017; Núñez et al., 2020). We obtained direct scores and subsequently calculated each subject’s scale in each of the measures (Gómez-Millán et al., 2017; Núñez et al., 2020).

#### State-Trait Anxiety Inventory

The Brazilian version (Biaggio and Natalício, 1979) of the State-Trait Anxiety Inventory (STAI-state, STAI-trait) (Spielberger et al., 1980) was used. This questionnaire is composed of two blocks (Form 1 and Form 2) of 20 statements, evaluated in a four-point Likert scale (1- absolutely not; 2- a little; 3- enough; 4- very much). Form 1—STAI-State, the state of anxiety reflects a transient reaction directly related to a situation of adversity that presents itself during a current moment. Form 2—STAI-Trait, refers to the individual’s propensity to deal with greater or lesser anxiety throughout their life. The Cronbach alphas of each scale were extracted to assess the questionnaires’ internal consistency, finding a Cronbach’s alpha of 0.960 for the STAI-E and Cronbach’s alpha of 0.705 STAI-T, indicating the instrument’s reliability (Cronbach and Warrington, 1951). The score is generated by the sum of the 20 items for each scale. Higher levels correspond to higher anxiety levels.

### Statistical Analysis

All analyses were performed utilizing SPSS software version 20.0 package (SPSS, Chicago, United States). Descriptive data are presented as percentage/absolute frequency, and factor analysis (principal components analysis method) was used to reduce a large number of variables into fewer factors of each anxiety group (state and trait) to verify which variables are associated with the COVID-19 pandemic anxiety state. This technique extracted the maximum common variance from all variables and put them into a common anxiety score associated with soccer athletes, coaches, and physical trainers. A significance level of *p* ≤ 0.05 was used.

## Results

Present results indicated that male soccer teams had higher state and trait anxiety than female soccer teams (Table 1).

**TABLE 1:** Descriptive state and trait anxiety analysis between female and male soccer teams.

Variables	Female (*n* = 99)	Male (*n* = 465)
State anxiety (index)	54.97 ± 9.43	57.65 ± 9.48*
Trait anxiety (index)	54.21 ± 5.74	55.76 ± 6.41

*Statistical difference is significant *p*≤0.05.

Statistical analysis indicated that state anxiety [*F*(1, 561) = 6.546; *p* = 0.011] and trait anxiety [*F*(1, 561) = 4.956; *p* = 0.026] in males on average is greater than in females. Comparing male and female soccer professionals and athletes according to gender, we found some factors that showed a significant difference that may influence participants’ anxiety during the pandemic COVID-19.

The results showed that the practice of physical activity had a more significant impact on soccer professionals than on athletes, which may indicate that because of the habituation of exercises, the effects are lower on the athletes’ anxiety control. As well as, in the perception of subjective performance, athletes accustomed to the practice did not feel good about performance during quarantine. For this reason, they are more susceptible to increased anxiety, indicating that mental training may be valid in this situation. When we analyze anxiety in the pandemic due to the pressure of international matches, male professionals have the highest rate, probably due to the uncertainties of contractual issues.

### Female Soccer Teams

The female group in relation to the level of education, have 2% Complete Elementary Education, 5.1% Incomplete Elementary Education, 9.1% Complete High School, 18.2% Incomplete High School, 14.1% Complete Higher Education, 9.1% Incomplete higher education, 35.4% Complete post-graduate and 7.1% Incomplete post-graduate. Marital status 33.3% Married, 8.1% divorced, and 58.60% single. Of these, 48.5% are from Rio de Janeiro, 14.1 São Paulo, 10.1 Brasilia and the remaining 27.3% from Bahia, Curitiba, Espírito Santo, Fortaleza, Minas Gerais, Paraiba, Piaui, Rio Grande do Sul, Rondônia and Santa Catarina. According to the length of service, 7.1% work with football for 1 year, 28.3% over 10 years, 8.1% over 20 years, 28.3% between 2 and 5 years, and 28.3% between 6 and 10 years. Regarding seminal training, 24.2% train 3x weeks, 22.2% 5x, 28.3% 6x, and the rest varies between 1 and 12 times per week. Regarding hours, 23.2% train 2 h per week and the rest varies between 1 and 18 h. During the pandemic, 23.2% exercised during social isolation (COVID-19), and 76.8% did not. Of these, 38.4% are football players, and 76.8 are football professionals.

### Male Soccer Teams

The male group in relation to the level of education, have 3.7% Complete Elementary Education, 4.7% Incomplete Elementary Education, 14.0% Complete High School, 15.4% Incomplete High School, 18.1% Complete Higher Education, 15.4% Incomplete higher education, 23.0% Complete post-graduate and 5.9% Incomplete post-graduate. Marital status 30.7% Married, 4.0% divorced, and 65.1% single. Of these, 37.4% are from Rio de Janeiro, 23.8% São Paulo, 8.3% Minas Gerais and the remaining 26.0% from Brasilia Bahia, Curitiba, Espírito Santo, Fortaleza, Paraiba, Piaui, Rio Grande do Sul, Rondônia and Santa Catarina. According to the length of service, 6.2% work with football for 1 year, 31.6% over 10 years, 11.2% over 20 years, 20.2% between 2 and 5 years, and 30.9% between 6 and 10 years. Regarding seminal training, 35.1% train 6x weeks, 22.5 5x, 16.0% 7x, and the rest varies between 1 and 12 times per week. Regarding hours, 18.1% train 2 h per week and the rest varies between 1 and 40 h. During the pandemic, 88.5% exercised during social isolation, and 11.5% did not. Of these, 42.9% are soccer players, and 57.1% are soccer professionals. Self-perceived performance during the pandemic COVID-19 is shown in Table 2.

**TABLE 2:** General performance in soccer, physical, group, the technical, tactical, psychological, and disciplinary self-perceived performance of female and male soccer teams.

Variables	Female (*n* = 99)		Male (*n* = 465)	
		
	Professional (*n* = 61)	Athletes (*n* = 38)	Professional (*n* = 261)	Athletes (*n* = 204)
Current performance	3.590 ± 0.989	3.711 ± 0.767	3.875 ± 0.891	3.760 ± 0.817
Current physical performance	3.984 ± 0.695	4.026 ± 0.592	3.900 ± 0.732	3.897 ± 0.758
Current individual performance	3.836 ± 0.840	3.842 ± 0.678	3.900 ± 0.723	3.784 ± 0.814
Current group performance	3.754 ± 1.059	3.868 ± 0.991*	3.935 ± 0.881	3.956 ± 0.795
Current technical performance	3.672 ± 0.723	4.053 ± 0.733*	3.935 ± 0.779	3.897 ± 0.718
Current tactical performance	3.902 ± 0.746	3.842 ± 0.754	4.015 ± 0.662	3.936 ± 0.743
Current psychological performance	3.656 ± 0.910	3.974 ± 0.914	3.805 ± 1.013	3.882 ± 0.990
Current disciplinary performance	3.754 ± 1.349	4.395 ± 1.027*	4.391 ± 0.744	4.309 ± 0.773

*Statistical difference is significant *p*≤0.05.

There was a statistical difference between the female groups when compared with professionals and athletes in the variables Current Technical Performance [*F*(1, 97) = 6.405; *p* = 0.013], Current Group Performance [*F*(1, 97) = 6.526; *p* = 0.012] and Current Disciplinary Performance [*F*(1, 97) = 6.282; *p* = 0.014], however, there was no difference between the female and male groups. There was no statistical difference in the male group when compared to professionals and players, as well as when compared between the male and female groups.

## Discussion

Present data demonstrated higher trait and state anxieties in male soccer teams than female soccer teams. In addition, athletes and professionals of soccer teams had differences in self-perceived performances—possibly impacted by anxiety during pandemic COVID-19. The results show that the athletes have a view that they have a medium or good physical, technical, and tactical performance so far, higher performance than the professionals’ self-perception about the same factors suggesting that individuals who work with soccer outside of field understand that the questions are exclusive to the athletes, regarding the perception of collective performance to date. Soccer professionals have a higher frequency than athletes, demonstrating a sense of collectivity and belonging to the group and measuring the team’s evolution in times of pandemics.

In contrast, athletes who do not play matches have a lower perception of collective performance. On the other hand, the perception of individual performance so far is relatively higher among athletes. They understand that their performance is good or very good, different from the result presented by soccer professionals who reported that their performance is bad. One hypothesis that can be raised in this case is that soccer professionals in confinement because of the quarantine have a self-perception that they are not useful in this period, associating the importance of their work only with the practical part, ignoring their role in periodization and monitoring of the training that are essential even if at a distance. Regarding the psychological performance factor, it can be noted that the professionals believe that their psychological performance is excellent, and the athletes report a poor psychological performance during the pandemic. Self-perception of the discipline so far has obtained higher numbers in athletes of both genders.

The present study demonstrated higher anxieties in male vs. female soccer teams; this could affect professional and athletic results and suggest future psychological interventions in each gender group. Previous articles in the literature investigated whether there were differences in anxiety in sport according to gender (Gonçalves and Belo, 2007; Munhóz, 2012; Correia and Rosado, 2019). The study by Gonçalves and Belo proposes to investigate competitive trait anxiety regarding gender, age group, experience in competitions, and type of sports (individual and collective) in young athletes. For this, 105 athletes from Paraiba, from various sports modalities, participated in the research (Futsal—14.3%; Handball- 5.7%, Synchronized swimming—9.5%; Swimming—33.3% and Volleyball—37.1%), with ages ranging from 11 to 20 years being the majority male (55.2%). The researchers used the Sport Competition Anxiety Test—SCAT to measure anxiety. As a result, there was a statistically significant difference in competitive trait anxiety concerning the gender of the participants. Another study that presented anxiety in sport was Munoz, which also found factors related to anxiety in women’s soccer. The study included 16 female soccer athletes aged between 14 and 20 years of the team of the Municipal Sports Department of the city of Bebedouro/SP. As a test, the Inventory of State Competition (ISC). The authors made statistical analyses with Pearson’s correlation test showed a significant inverse relationship between anxiety levels and the time of experience with soccer (rp = –0.634; *p* = 0.008) and the number of championships participated (rp = –0.528; *p* = 0.036). It can be concluded with this study that athletes with more practice time and a greater number of participations in soccer championships have lower levels of anxiety. The research of Correia and Rosado also aimed to investigate athletes’ sports anxiety regarding differences in gender and sport played.

An application of structural equation modeling was made with 601 Portuguese athletes. 172 (28.6%) were female, and 429 (71.4%) were male. They competed in various individual (e.g., athletics, climbing, orienteering, surfing, swimming, tennis; 42.6%) and team sports (e.g., basketball, handball, rugby, soccer, volleyball; 57.4%). Significant differences were detected between male and female athletes and between individual and team sports. Female and individual sports athletes presented higher levels of general sports anxiety. These results also show greater female anxiety. However, like our study, the difference in the sample is significant between genders. The greater anxiety in individual sports corroborates the hypothesis that the absence of a group can increase anxiety. Athletes experienced this reality during the pandemic.

The study has some limitations that can be investigated in future research. As a limitation, we can mention that the study does not directly compare the female and male gender due to the great difference between the sample. The collection was carried out at a single moment in the pandemic. It could have been repeated in other parts of the COVID-19 evolution curve and at the end of the pandemic to compare the moments in the timeline. The authors suggest that further studies should be carried out to compare athletes who have a medical report for generalized anxiety since, in a self-perceived survey, the result may be underestimated or overestimated by the participants.

The findings of this study highlight the effects on anxiety in athletes and soccer professionals during the first wave of the COVID-19 pandemic in Brazil and the difference in anxious behaviors and factors that imply greater differences between genders in sport. The results demonstrate that there are significant differences between professionals and athletes within each gender. As the field progresses, further studies should seek to verify possible group differences according to clinical diagnoses of anxiety vs. self-report symptoms, as there may be bias in self-report assessments, whether in relative over reporting or underreporting (Reardon et al., 2019). Analyzing the mental health of genders in soccer is important to understand how each one has dealt with the pandemic situation, and further research should be carried out at another time to compare the chronological periods.

## Conclusion

Present research aimed to compare the factors that can increase trait and state anxiety among professionals and soccer players according to the influence of gender during the COVID-19 pandemic. Our data demonstrated higher trait and state anxieties in male soccer teams than female soccer teams. In addition, athletes have better sociodemographic self-perception than professionals of soccer. Soccer professionals demonstrated a sense of collectivity and belonging to the group and being able to measure the team’s evolution in times of pandemic. In practice, it is necessary to observe the needs of each of the groups so that the intervention in the psychological part is not a generalist. Future research should be conducted to examine the gendered outcomes of athletes and soccer professionals within the clinical range for anxiety disorders during the COVID-19 pandemic.

## Data Availability Statement

The original contributions presented in the study are included in the article/supplementary material, further inquiries can be directed to the corresponding author/s.

## Ethics Statement

The studies involving human participants were reviewed and approved by the Federal University of Rio de Janeiro (no. 13846919.8.0000.5257). The patients/participants provided their written informed consent to participate in this study.

## Author Contributions

All authors of the present study worked equivalently in all phases of this research.

## Conflict of Interest

The authors declare that the research was conducted in the absence of any commercial or financial relationships that could be construed as a potential conflict of interest.

## Publisher’s Note

All claims expressed in this article are solely those of the authors and do not necessarily represent those of their affiliated organizations, or those of the publisher, the editors and the reviewers. Any product that may be evaluated in this article, or claim that may be made by its manufacturer, is not guaranteed or endorsed by the publisher.
